# Characterization of ovarian cancer cells and tissues by Fourier transform infrared spectroscopy

**DOI:** 10.1186/s13048-018-0434-8

**Published:** 2018-08-02

**Authors:** Lei Li, Xiaoning Bi, Hengzi Sun, Simiao Liu, Mei Yu, Ying Zhang, Shifu Weng, Limin Yang, Yanan Bao, Jinguang Wu, Yizhuang Xu, Keng Shen

**Affiliations:** 1 Department of Obstetrics and Gynecology, Peking Union Medical College Hospital, Chinese Academy of Medical Sciences & Peking Union Medical College, No. 1 Shuai Fu Yuan, Eastern District, Beijing, 100730 China; 2 Beijing National Laboratory for Molecular Sciences, State Key Laboratory for Rare Earth Materials Chemistry and Applications, College of Chemistry and Molecular Engineering, Peking University, No. 202 Chengfu Road, Haidian District, Beijing, 100871 China; 3 State Key Laboratory of Nuclear Physics and Technology, Institute of Heavy Ion Physics, School of Physics, Peking University, No. 202 Chengfu Road, Haidian District, Beijing, 100871 China

**Keywords:** Ovarian cancer, Fourier transform infrared (FTIR) spectroscopy, Tumor heterogeneity, Clinical diagnosis

## Abstract

**Background:**

Ovarian cancer is the most lethal of gynecological malignancies. Fourier Transform Infrared (FTIR) spectroscopy has gradually developed as a convenient, inexpensive and non-destructive technique for the study of many diseases. In this study, FTIR spectra of normal and several heterogeneous ovarian cancer cell lines as well as ovarian cancer tissue samples were compared in the spectral region of 4000 cm^− 1^ - 600 cm^− 1^.

**Methods:**

Cell samples were collected from human ovarian surface epithelial cell line (HOSEpiC) and five ovarian cancer cell lines (ES2, A2780, OVCAR3, SKOV3 and IGROV1). Validation spectra were performed on normal and cancerous tissue samples from 12 ovarian cancer patients. FTIR spectra were collected from a NICOLET iN10 MX spectrometer and the spectral data were analyzed by OMNIC 8.0 software.

**Results:**

Spectral features discriminating malignant tissues from normal tissues were integrated by cell line data and tissue data. In particular changes in cancerous tissues, the decrease in the amount of lipids and nucleic acids were observed. Protein conformation and composition were also altered in some cancer cells. The band intensity ratio of 1454/1400 was higher in normal cells/tissues and lower in cancer cells/tissues.

**Conclusion:**

The spectral features revealed the important molecular characteristics about ovarian cancer cells/tissues. These findings demonstrate the possible diagnostic use of FTIR spectroscopy, providing the research model and evidences, and supporting the future study on more tissue samples to establish a data bank of spectra features for the possible discrimination of ovarian cancers.

## Background

Ovarian cancer is associated with the worst prognosis among gynecological malignancies, accounting for more than 150,000 deaths annually worldwide [1]. Most patients with advanced stages of ovarian cancer result in five-year survival rates less than 30% [2]. Ovarian cancer includes several heterogeneous subtypes with different clinical phenotypes, molecular features and prognosis. About 90 % are epithelial ovarian cancers (EOC) [3], which is traditionally subtyped as serous, endometrioid, clear cell and mucinous. Among them, serous ovarian cancer accounts for about 70% of EOC [4]. High-grade serous ovarian cancer (HGSOC), the most malignant subtype, is responsible for 90% of serous tumors [5]. Developing methods for detecting ovarian cancer at the early stage and illuminating the molecular mechanism underlying this tumor heterogeneity would provide us new insights into ovarian cancer and lead to more effective diagnostic approaches.

Currently, there are two approaches available for diagnosing ovarian cancer patients. One is the detection of the blood-derived biomarkers. The frequently used biomarker is serum Cancer Antigen 125 (CA-125) [6]. However, limited specificity exists in this method as CA-125 level also rises in some other types of cancers and also fluctuates in premenopausal women during menstrual cycle [7]. The other method is to provide detailed images of ovaries through imaging techniques such as Magnetic Resonance Imaging (MRI), Doppler Ultrasound and Computed Tomography (CT). However, ultrasound shows a poor accuracy in diagnosing diseases at early stages [8]. It is obvious that these current methods have some limitations. Therefore new insights are needed to identify novel methods for detecting and categorizing ovarian cancer patients.

Vibrational spectroscopy is one kind of the bio-analytical methods that increasingly shows significant potential to provide a novel diagnostic tool to distinguish normal and pathological tissues [9]. Fourier-transform infrared (FTIR) spectroscopy, in particular, has been utilized in the past several decades [10–12]. It has the advantage of convenience and non-destruction to detect tumors with minimal sample preparations, and also allows the investigation of both qualitative and quantitative assesses of certain components [13, 14].

Many studies by this technique have been focused on several cancer cells or tissues, such as endometrial [15], cervical [16], breast [17], lung [18] and brain cancers [19]. Few studies have screened ovarian cancer by FTIR. Gajjar et al. [20], Owens et al [21] and Lima et al [22] have respectively examined blood plasma or serum of ovarian cancer by attenuated total reflection Fourier-transform infrared (ATR-FTIR) spectroscopy coupled with other selection methods. Mehrotra et al [23] analyzed FTIR data of the post surgical tissue sections of ovarian cancer and discovered some particular changes in the spectral regions of protein, nucleic acid and lipid, using 12 samples without classifying the cancer subtype. Theophilou et al [24] provided a novel approach in discriminating normal, borderline and malignant ovarian tissues. The authors used ATR-FTIR spectroscopy combined with three chemometric methods followed by linear discriminant analysis. However, there were little common insights in these few studies, as they explored on different aspects. In the present study, we fundamentally focused on EOC with respect to its normal and several heterogeneous cancer cell line models using FTIR technique and confirmed the results by several tissue samples, providing proof of principal that there were differences between EOC and healthy donor epithelium and supporting the future study to move to more clinical tissue samples to establish a data bank of spectra features.

## Methods

### Cell culture

The normal cell line used in this study was human ovarian surface epithelial cell line (HOSEpiC) obtained from ScienCell Research Laboratories (San Diego, CA). The ovarian cancer cell lines used in this study were ES2, A2780, OVCAR3, SKOV3 and IGROV1. Among them, ES2, A2780 and SKOV3 were purchased from the Cell Support Center, Institute of Basic Medical Science, Chinese Academy of Medical Sciences; OVCAR3 and IGROV1 were purchased from the NIH cell bank. OVCAR3 originates from the malignant ascites of a patient with ovarian adenocarcinoma [25], possesses characteristics of HGSOC [26]. ES2 is a recognized ovarian clear cell carcinoma cell line [27]. IGROV1, originating from an ovarian carcinoma of a 47-year-old woman, is an ovarian adenocarcinoma cell line with multiple differentiations, and endometrioid is its major part [28]. A2780 and SKOV3 both have been widely used as models for HGSOC. However, recent studies demonstrated that they carried some characteristics of endometrioid/clear cell ovarian carcinomas [26].

HOSEpiC, A2780, IGROV1 and OVCAR3 cells were incubated in Roswell Park Memorial Institute (RPMI)-1640 medium (HyClone, Logan, Utah, USA) supplemented with 15, 10, 10 and 20% heat-inactivated fetal bovine serum (FBS; Gibco, Carlsbad, CA, USA), respectively, at 37 °C in 5% carbon dioxide. ES2 and SKOV3 cells were cultured in McCoy’s 5A medium (HyClone) containing 10% FBS at 37 °C in 5% carbon dioxide. The cells were subcultured when they reached approximately 80% confluence, and harvested at almost the same life time after two passages.

### Cell preparation for spectroscopy

Cells were trypsinized and washed twice in saline, suspended and centrifuged at 1000 rpm for 5 min. Supernatant was then removed gently and cells were stored at − 80 °C until removal for experiments.

### Tissue collection and preparation for spectroscopy

Tissue specimens from 12 cases of EOC were obtained from Peking Union Medical College Hospital, Chinese Academy of Medical Sciences & Peking Union Medical College. Informed consents have been taken before surgery. Cancer tissue and their corresponding normal tissue samples were collected respectively from 10 histologically serous cancer and 2 clear cell cancer patients. Tissue samples were frozen at − 80 °C until removal for spectral scanning.

### FTIR spectroscopy

FTIR spectra of cell and tissue samples were collected using a NICOLET iN10 MX (Thermo Scientific, Waltham, MA, USA) spectrometer, equipped with a KBr/Ge beam splitter and a mercury cadmium telluride (MCT) detector. For each spectrum, 16 scans were performed at the resolution of 4 cm^− 1^ between 4000 cm^− 1^ and 600 cm^− 1^. The spectrum was then baseline corrected and normalized from 0 to 1 so as to have better comparison for the intensity.

### Analysis of FTIR data

Second Derivative (DII) and Curve Fitting (Gaussian algorithm) procedures were performed to identify the precise position and absorbance of specific bands. By using OMNIC 8.0 software (Thermo Fisher Scientific), Curve Fitting was conducted on spectra in the range of 1700–1600 cm^− 1^ after two points baseline linear fitted. In order to determine the underlying component bands, the number of peaks as well as their positions was identified based on DII results, resulting in the optimal reconstructed curve (residual close to zero). Positions and percentages of band areas were obtained for every component peak.

### Subtraction spectra

In order to further identify spectral variations between normal cells and ovarian cancer cells, the spectrum of ovarian surface epithelial cell line (HOSEpiC) was subtracted by the spectrum of each of the ovarian cancer cell lines, respectively. Thus we obtained the “subtraction spectra”. All of the subtraction spectra were calculated using fully preprocessed spectra, that were baseline corrected and normalized from 0 to 1.

### Statistical analysis

The percentages of band areas are presented as mean ± SEM. For statistical comparisons among multiple cell lines, one-way ANOVA test was used by SPSS 17.0 software (Chicago, Illinois, USA) with a significance set at *P* < 0.05 for at least three times. For statistical comparisons of spectra intensities between normal and malignant tissues at specific bands, a Wilcoxon paired signed-rank test with a significant level of 0.05 was used by SPSS 17.0 software.

## Results

### FTIR spectral analysis for normal and ovarian cancer cell lines

**Fig. 1:**
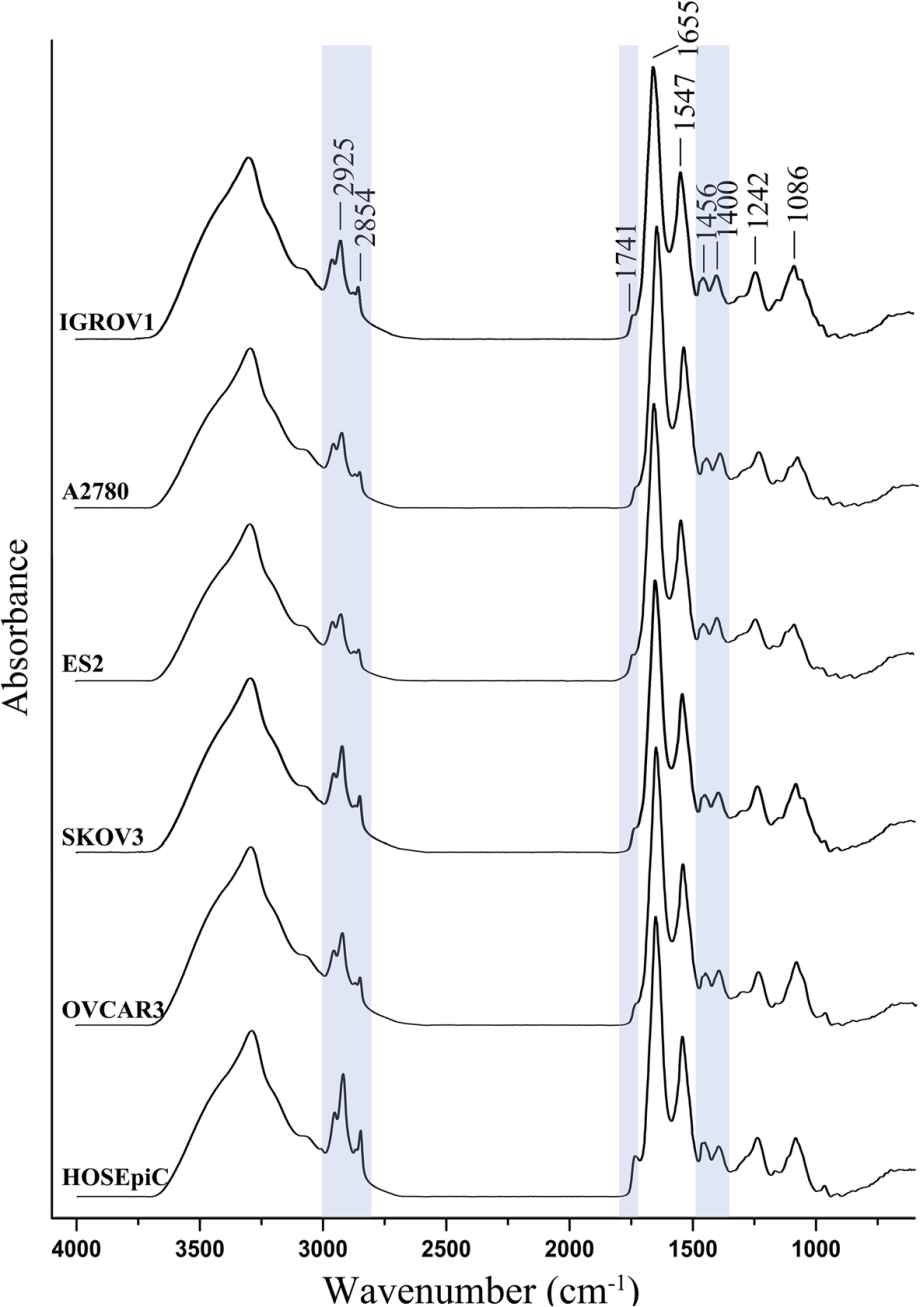
Representative FTIR spectra of ovarian cancer cells and normal cells. FTIR spectra of the five ovarian cancer cell lines (IGROV1, A2780, ES2, SKOV3 and OVCAR3) and the normal human ovarian surface epithelial cell line (HOSEpiC) were presented in the region 4000–600 cm^− 1^. The apparent differences were indicated in blue box

This study first focused on the biochemical differences between several heterogeneous ovarian cancer cell lines (ES2, A2780, OVCAR3, SKOV3 and IGROV1) and the normal human ovarian surface epithelial cell line (HOSEpiC) by FTIR spectroscopy. For each cell line, a typical spectrum was presented (Fig. 1). The apparent spectral differences were observed, above all in the C-H stretching region of fatty acids in cell membranes (3000–2800 cm^− 1^), in the ester C = O stretching of phospholipids (1800–1700 cm^− 1^) and in the C-H bending vibrations of amino acid side chains and some lipids (1500–1300 cm^− 1^) [29]. Relative band intensities, which could be considered as spectral features for normal and cancer cells, were calculated. Second Derivative (DII) and Curve Fitting procedures of 1700–1600 cm^− 1^ were used to investigate Amide I of protein, and subtraction spectra were performed in the region of 4000–600 cm^− 1^ for additional comparison of each ovarian cancer cells versus normal cells.

### Comparison of the relative intensities of the specific bands for normal and ovarian cancer cell lines

**Fig. 2:**
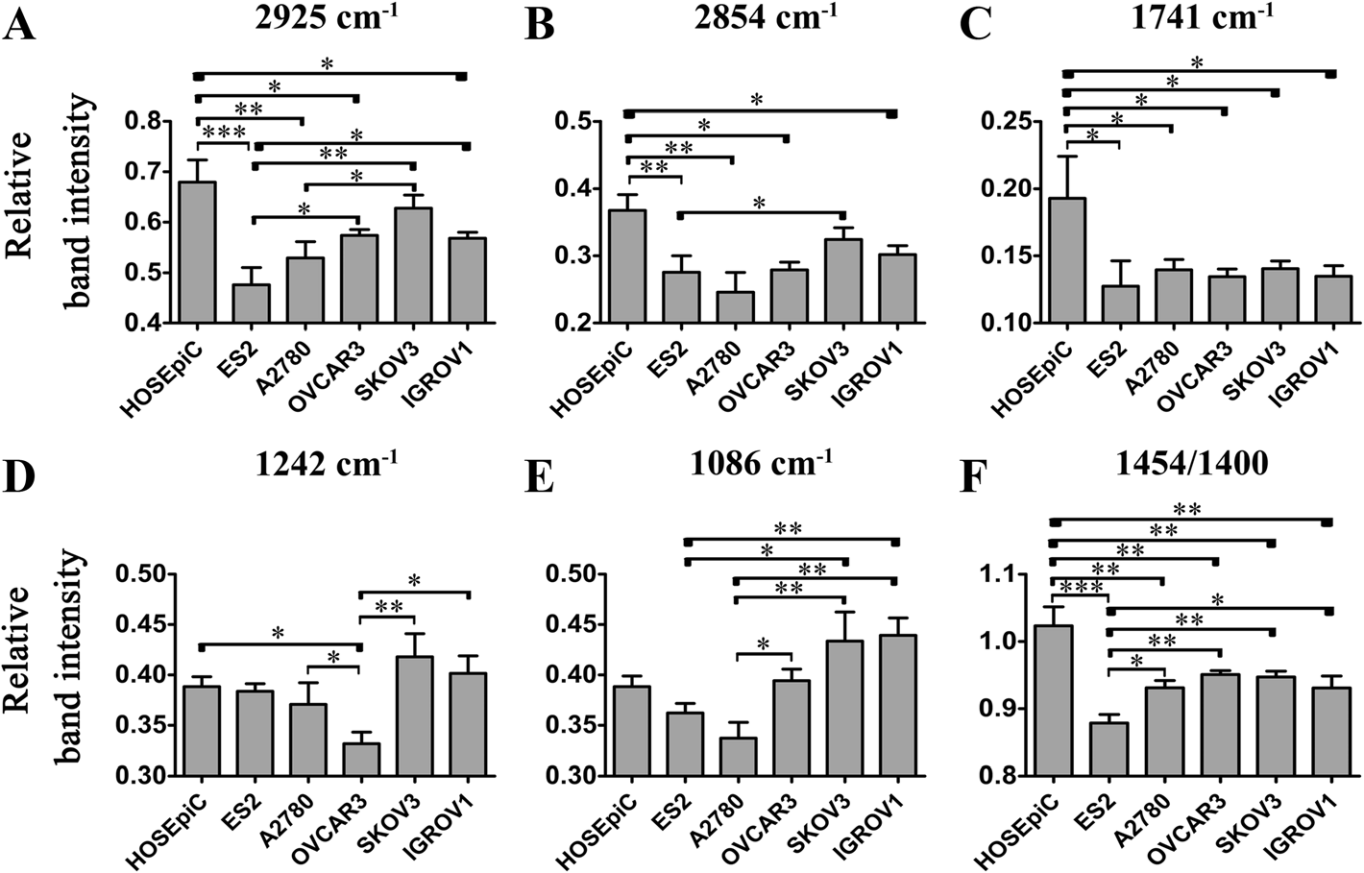
Relative band intensitie of normal cells and ovarian cancer cells. Error bars represent the SEM, *n* = 3 (*: *P* < 0.05. **: *P* < 0.01. ***: *P* < 0.001)

**Fig. 3:**
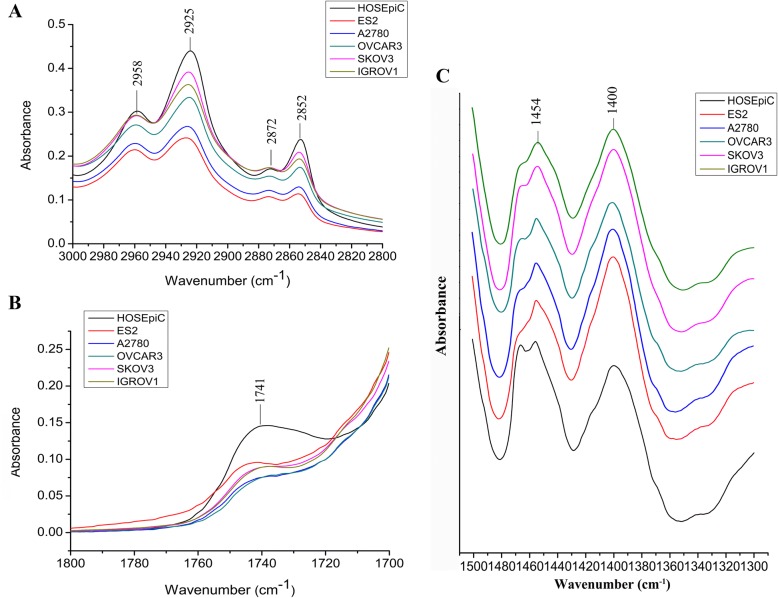
FTIR spectra in the region 3000–2800 cm^− 1^ (**a**), 1800–1700 cm^− 1^ (**b**) and 1500–1300 cm^− 1^ (**c**)

Several spectral markers for cancer cell lines differed considerably from normal cell line, as shown in Fig. 2. The band signals at 2958 cm^− 1^ and 2925 cm^− 1^ (ν_asym_ CH_3_ and CH_2_), 2872 cm^− 1^ and 2854 cm^− 1^ (ν_sym_ CH_3_ and CH_2_) are characteristics of alkyl chains mainly present in lipids [30]. After analyzing the relative band intensities, lower amounts of the relative intensities of 2925 cm^− 1^ and 2854 cm^− 1^ were found in the five cancer cells as compared to HOSEpiC cells, respectively (Fig. 2a, b), consisting with their corresponding FTIR spectra in the region of 3000–2800 cm^− 1^ (Fig. 3a). The differences were significant for ES2, A2780, OVCAR3 and IGROV1 vs. HOSEpiC, respectively, indicating a lower amount of lipids present in the four cancer cells. Moreover, ovarian cancer cell lines also showed heterogeneous characteristics between themselves. ES2 showed lower relative intensity at 2925 cm^− 1^ than that of OVCAR3, SKOV3 and IGROV1, respectively. The relative intensity at 2925 cm^− 1^ in A2780 was significantly lower from that in SKOV3, while the relative intensity at 2854 cm^− 1^ in ES2 was significantly lower from that in SKOV3 (Fig. 2a, b).

The absorption bands at 1242 cm^− 1^ (*ν*_as_PO_2_^−^) and 1086 cm^− 1^ (*ν*_s_PO_2_^−^) were attributed to asymmetric and symmetric phosphodiester vibrations of nucleic acids [31]. The relative intensity at 1242 cm^− 1^ in OVCAR3 was lower than that in HOSEpiC, A2780, SKOV3 and IGROV1, respectively (Fig. 2d). A2780 showed lower relative intensity at 1086 cm^− 1^ than that of OVCAR3, SKOV3 and IGROV1, while ES2 showed lower relative intensity at 1086 cm^− 1^ than that of SKOV3 and IGROV1 (Fig. 2e). The differences were all significant. These findings also suggested heterogeneous characteristics between ovarian cancer cells.

In the absorption signals between 1800 and 1700 cm^− 1^, the prominent band intensity difference was found around 1741 cm^− 1^, which was attributed to ester C = O stretching of phospholipids [32, 33]. It is obvious that the signal at 1741 cm^− 1^ was enhanced in HOSEpiC cells than in all of the ovarian cancer cells (Fig. 2c, Fig. 3b), indicating increased lipids and esterified components in cell membranes in normal ovarian cells than in cancerous cells. This result was in corroboration with the previous finding in breast cancer cells that the peak at 1741 cm^− 1^ increased progressively with the increasing 5-fluorouracil dose in MCF-7 cells (human breast adenocarcinoma cell line) [34]. As 5-fluorouracil overtly inhibited tumor cell proliferation in a concentration dependent manner, cells with the high level of proliferation showed the lower peak at 1741 cm^− 1^, correspondence with our result that the malignant ovarian cancer cells exhibited lower intensities at 1741 cm^− 1^ than normal ovarian cells.

The bands at 1454 and 1400 cm^− 1^ usually attribute to the C-H bending vibrations of various amino acid side chains and some lipids, etc. [29]. They are critical bands to distinguish normal and malignant tissues, and the precise assignments are not clear [35]. It is obvious to see that the band height at 1454 cm^− 1^ was always lower than that at 1400 cm^− 1^ in all of the ovarian cancer cell lines (*I*_1454_/*I*_1400_ ˂ 1). In HOSEpiC cells, comparison of the heights of the two bands were apparently opposite (*I*_1454_/*I*_1400_ ˃ 1). Besides, ES2 exhibited the lowest ratio of 1454/1400 among other cells (Fig. 2f, Fig. 3c).

### Curve fitting of protein amide I in normal and ovarian cancer cell lines

**Fig. 4:**
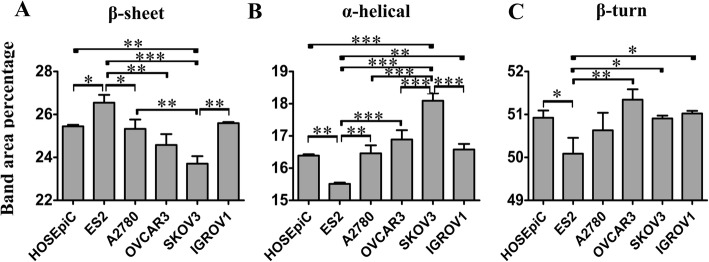
Percentage of secondary structures of protein Amide I in normal and ovarian cancer cells. (**a**) β-sheet, (**b**) α-helix, (**c**) β-turn structures. Error bars represent the SEM, *n* = 3 (*: *P* < 0.05. **: *P* < 0.01. ***: *P* < 0.001)

The region between 1700 cm^− 1^ and 1500 cm^− 1^ was usually assigned to protein absorption with dominant bands at ~ 1655 cm^− 1^ (Amide I) and ~ 1547 cm^− 1^ (Amide II) [36]. After the Curve Fitting procedure, Amide I of the cells was analyzed respectively and split into a series of bands. In general for Amide I, α-helical structures have a band at 1653 ± 4 cm^− 1^; β-sheet structures occurs between 1620 and 1640 cm^− 1^; β-turn structures are usually found at 1660–1680 cm^− 1^ [37]. The percentage of the component band areas, representative of their relative amounts, was calculated (Table 1). The band areas of β-sheet structures in HOSEpiC were significantly lower than that in ES2 and higher than that in SKOV3 [(25.45 ± 0.07) % vs. (26.55 ± 0.36) %, (25.45 ± 0.07) % vs. (23.71 ± 0.35) %], while the band areas of α-helical structures in HOSEpiC were significantly higher than that in ES2 and lower than that in SKOV3 [(16.39 ± 0.04) % vs. (15.51 ± 0.04) %, (16.39 ± 0.04) % vs. (18.09 ± 0.23) %]. HOSEpiC also had a higher band area of β-turn structures than ES2 [(50.92 ± 0.17) % vs. (50.09 ± 0.37) %]. There also seemed significant differences between normal and some cancer cells and differences among the different types of ovarian cancer cells (Fig. 4, Table 1). These findings indicated that the secondary structure of protein had been altered in some but not all types of cancer cells, probably attributed to cell specificity.

**Table 1:** Percentage of component band areas after Curve Fitting procedure in the region 1700–1600 cm^− 1^

Position	Area (%)						Assignment
	HOSEpiC	ES2	A2780	OVCAR3	SKOV3	IGROV1	
1629	9.46 ± 0.12	9.45 ± 0.02	9.66 ± 0.15	9.40 ± 0.04	8.91 ± 0.17	9.43 ± 0.12	β-sheet
1639	15.99 ± 0.18	17.10 ± 0.35	15.67 ± 0.28	15.18 ± 0.47	14.80 ± 0.17	16.16 ± 0.17	β-sheet
**β-sheet SUM**	**25.45 ± 0.07**	**26.55 ± 0.36**	**25.33 ± 0.43**	**24.58 ± 0.50**	**23.71 ± 0.35**	**25.59 ± 0.06**	**β-sheet SUM**
**1649**	**16.39 ± 0.04**	**15.51 ± 0.04**	**16.46 ± 0.25**	**16.89 ± 0.29**	**18.09 ± 0.23**	**16.58 ± 0.17**	**α-helical**
1657	22.86 ± 0.30	21.09 ± 0.35	22.34 ± 0.06	22.44 ± 0.24	23.46 ± 0.11	23.64 ± 0.11	β-turn
1667	9.38 ± 0.16	9.82 ± 0.29	9.66 ± 0.08	9.53 ± 0.09	9.60 ± 0.15	9.35 ± 0.15	β-turn
1674	5.13 ± 0.10	5.66 ± 0.06	5.31 ± 0.15	5.05 ± 0.05	4.70 ± 0.05	4.77 ± 0.04	β-turn
1681	13.55 ± 0.13	13.52 ± 0.25	13.32 ± 0.58	14.32 ± 0.44	13.15 ± 0.06	13.26 ± 0.07	β-turn
**β-turn SUM**	**50.92 ± 0.17**	**50.09 ± 0.37**	**50.63 ± 0.41**	**51.34 ± 0.24**	**50.91 ± 0.07**	**51.02 ± 0.06**	**β-turn SUM**
1693	7.24 ± 0.20	7.85 ± 0.03	7.58 ± 0.23	7.19 ± 0.03	7.29 ± 0.05	6.81 ± 0.18	

Bold texts indicate the area percentages of β-sheet, α-helical and β-turn structures, respectively

### Subtraction spectrum of the ovarian cancer cells vs. normal cells

**Fig. 5:**
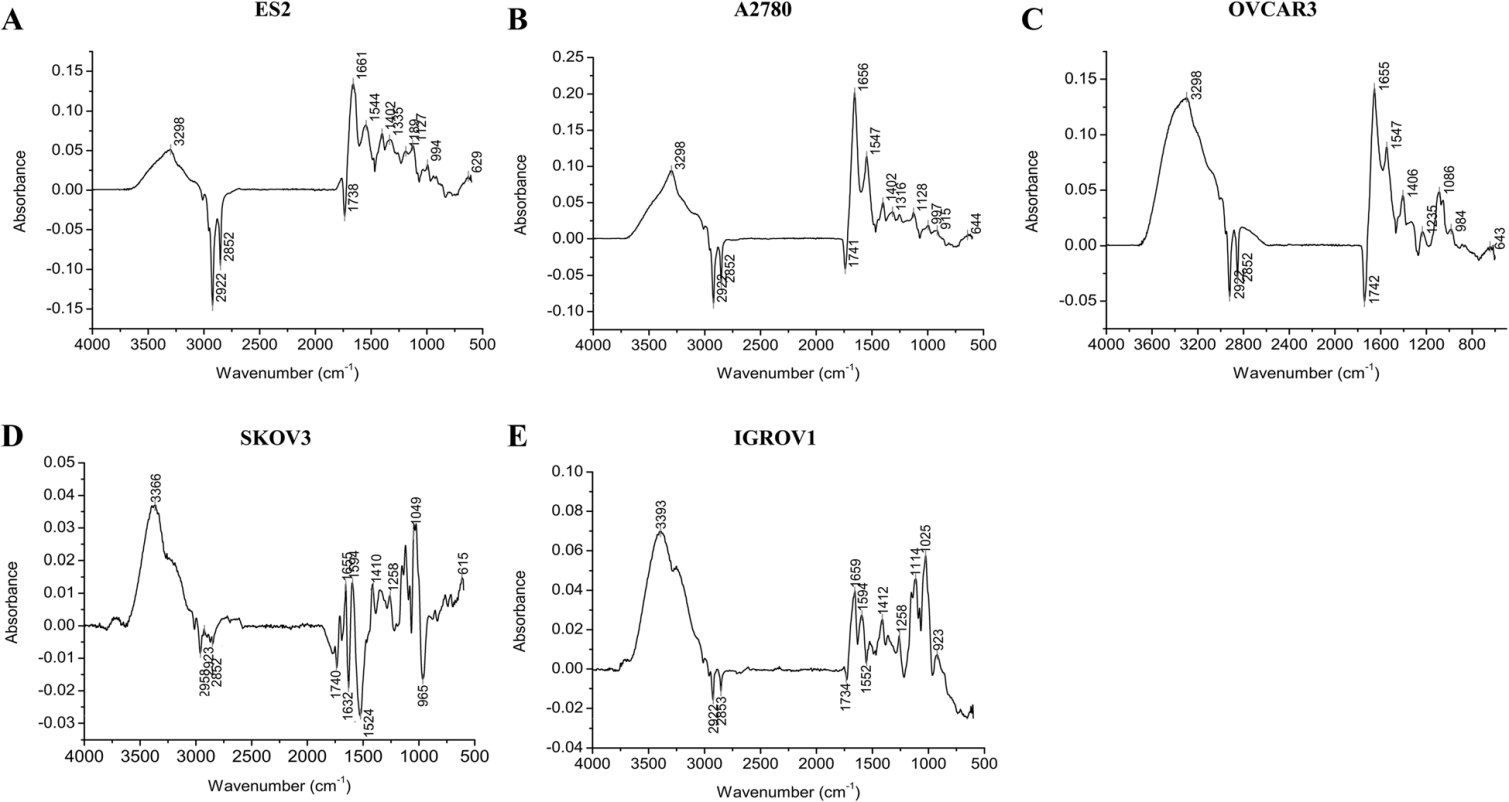
Subtraction spectra of each of the ovarian cancer cells vs. normal cells. **a** ES2 vs. HOSEpiC, (**b**) A2780 vs. HOSEpiC, (**c**) OVCAR3 vs. HOSEpiC, (**d**) SKOV3 vs. HOSEpiC, (**e**) IGROV1 vs. HOSEpiC

In order to further indentify the differences between normal cells and ovarian cancer cells, subtraction spectrum was performed for each of the cancer cell line vs. HOSEpiC, respectively. In subtraction spectra, the differences between the two samples in details were observed visibly. For example, if we have A and B spectra, adjusting subtraction factor using subtraction spectrum is performed, and the positive and negative bands were clearly observed. A-B, the same components of A and B were subtracted out; positive bands represent the part A > B, while negative bands represent the part B > A [38, 39]. As showing in Fig. 5, positive bands, indicating higher amounts, were seen in the region 3600–3000 cm^− 1^ (ν_N-H_ of protein) as well as at the bands around 1655 cm^− 1^, 1548 cm^− 1^ and 1406 cm^− 1^ in all of the ovarian cancer cells. Negative bands, indicating lower amounts, were seen at the bands around 2922 cm^− 1^, 2852 cm^− 1^ and 1741 cm^− 1^ in ES2, A2780, OVCAR3 and IGROV1; negative bands for the subtraction spectrum of SKOV3 vs. HOSEpiC were seen at 2958 cm^− 1^, 2852 cm^− 1^ and 1741 cm^− 1^. These were consistent with our above results that lower amounts in lipids (3000–2800 cm^− 1^) were found in ovarian cancer cells as compared to HOSEpiC cells, and that the signal at 1741 cm^− 1^ was enhanced in HOSEpiC cells than in ovarian cancer cells. Increased amount at the band ~ 1406 cm^− 1^ also indicated decreased ratio of 1454/1400 in ovarian cancer cells. These findings also suggested that ovarian cancer cells might have higher amount of protein (3600–3000 cm^− 1^, 1655 cm^− 1^ and 1548 cm^− 1^) than normal cells. As proteins play crucial role in the physiological processes, the alteration of conformation and content of proteins in cancer cells may indicate a diversified and impending energy demands for the metabolism of malignant cells.

### FTIR spectra analysis of normal and ovarian cancer tissues

**Fig. 6:**
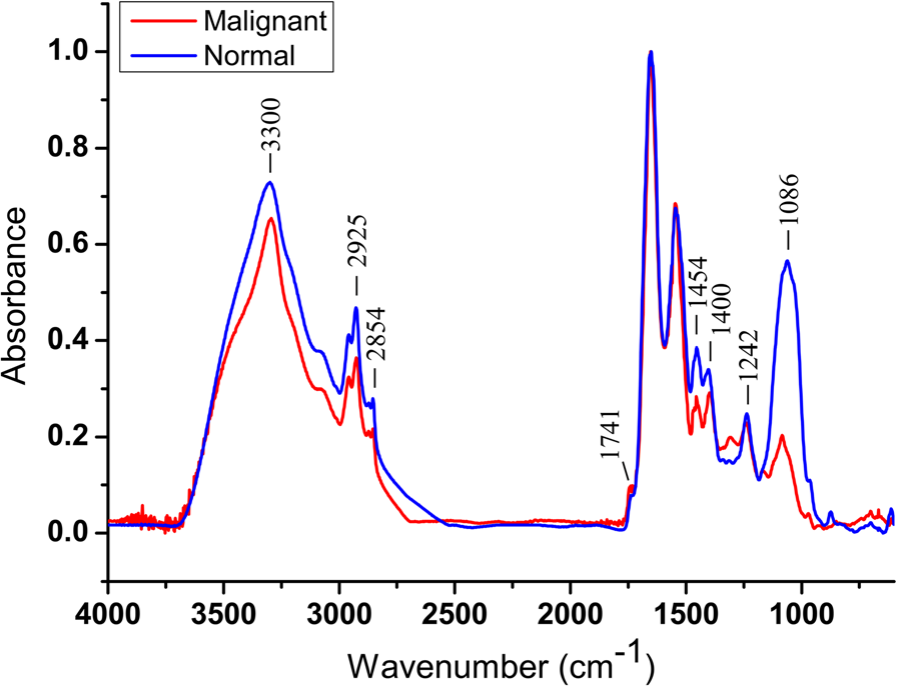
Representative FTIR spectra of normal and malignant ovarian tissue

To further confirm the data derived from ovarian cancer cell lines vs. HOSEpiC cell line, the FTIR spectra of normal and ovarian cancer tissues from 12 patients were analyzed. There were significant differences between normal and cancerous tissues identified in the regions 3000–2800 cm^− 1^, 1500–1300 cm^− 1^ and 1300–900 cm^− 1^ (Fig. 6).

**Fig. 7:**
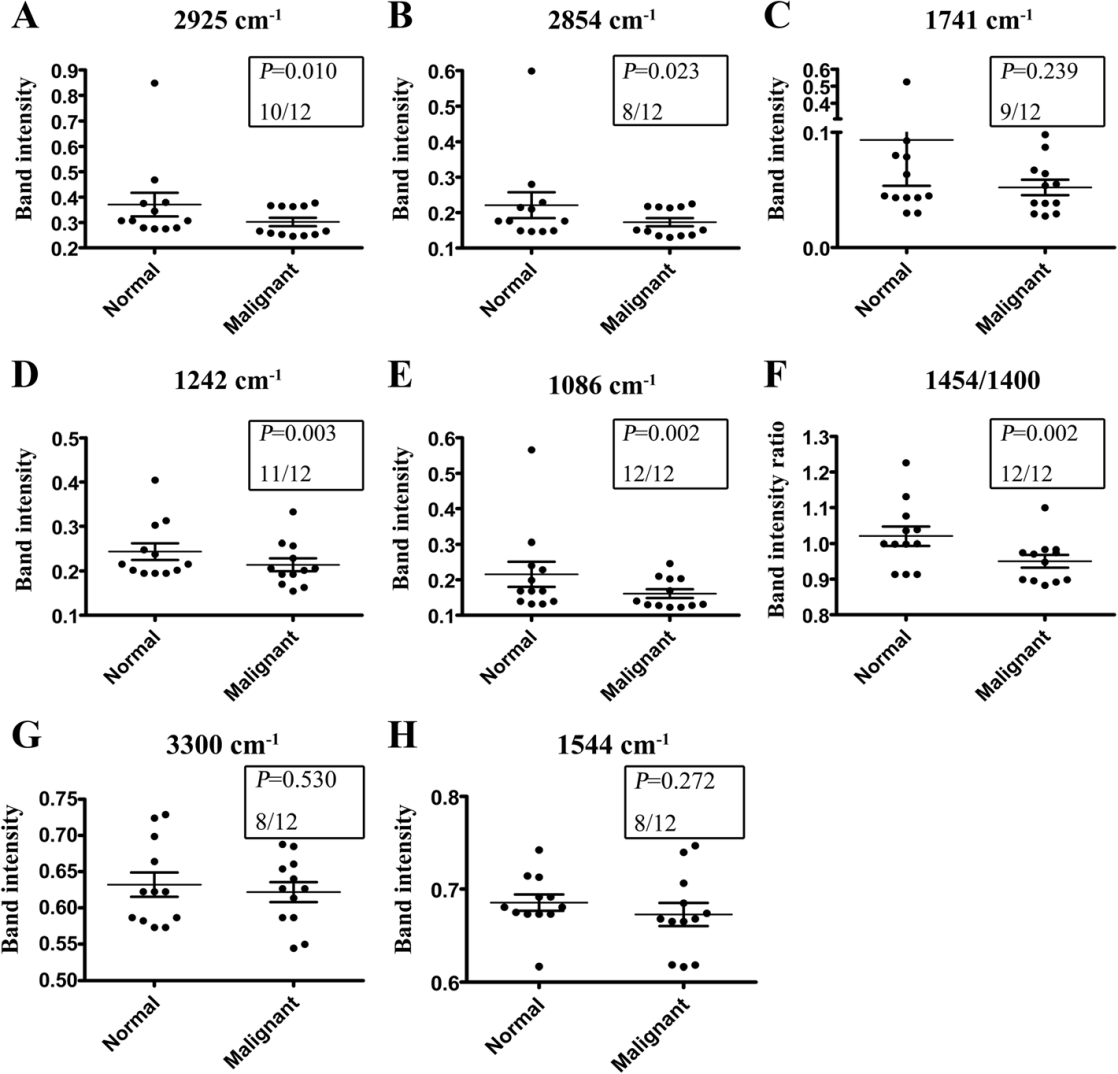
Comparison of the specific band intensities for normal and malignant ovarian tissues. Error bars represent the SEM. *P* value and the numbers of patients with *I*_normal_ > *I*_malignant_ at each specific bands (n/12) were indicated in boxes

Malignant tissues showed lower absorption signals in 3000–2800 cm^− 1^. The relative intensities of 2925 cm^− 1^ and 2854 cm^− 1^ were lower in malignant tissues and higher in normal tissues (Fig. 6); the differences were significant (*P* = 0.010, 0.023, respectively). Moreover, ten and eight ovarian cancer patients respectively showed lower absorption levels of 2925 cm^− 1^ and 2854 cm^− 1^ in malignant tissues (Fig. 7a, b), suggesting a decreased amount of lipids in malignant tissues. These results were consistent with the above cell line data.

For the absorption signal at around 1741 cm^− 1^, there was no significant difference (*P* = 0.239) between normal and malignant tissues, although there were still 9 in 12 patients showed lower signals at this peak in malignant tissues (Fig. 7c). Cell line data showed no significant differences between HOSEpiC and other five cancer cell lines at the band intensities of 1242 cm^− 1^ and 1086 cm^− 1^, however, malignant tissues showed significantly lower intensities at 1242 cm^− 1^ and 1086 cm^− 1^ than normal tissues (*P* = 0.003, 0.002), and almost all the cancerous samples had the same decrease (11/12, 12/12; Fig. 7d, e). These findings indicated lower amount of nucleic acids in malignant tissues, and 1242 cm^− 1^ and 1086 cm^− 1^ might have been new spectra signatures to distinguish normal and ovarian cancer tissues.

The relative band intensity ratios of 1454/1400 were significantly lower in malignant tissues than normal tissues in all of the ovarian cancer patients (*P* = 0.002; 12/12). Cell line data above concluded that the ratios were *I*_1454_/*I*_1400_>1 for normal cells and *I*_1454_/*I*_1400_<1 for cancer cells, however, there were 9 patients with *I*_1454_/*I*_1400_ ≥ 1 in normal tissues and 11 patients with *I*_1454_/*I*_1400_<1 in malignant tissues (Fig. 7f). Not all of the tissues coincided with the cell line results. Integrating the cell line data with the tissue data, it was undoubtedly to conclude that normal tissues showed higher 1454/1400 ratios than its corresponding malignant tissues.

Cell line data analyzed that ovarian cancer cell lines might have higher amount of protein than normal cells, however, the results of the tissue samples were the opposite. Most patients exhibited lower signal levels of 3300 cm^− 1^ and 1544 cm^− 1^ in malignant tissues (Fig. 7g, h), indicating that the spectra feature of protein was debatable and needed further investigation.

## Discussion

This study investigated the spectroscopic method to the identification of ovarian cancer. FTIR spectra were first analyzed on normal human ovarian surface epithelial cell line (HOSEpiC) and ovarian cancer cell lines (ES2, A2780, OVCAR3, SKOV3 and IGROV1) at the molecular level in order to a better understanding of their ingredients and contents. The analysis was performed on the spectral ranges of CH_2_, CH_3_ and C = O stretching modes of lipids, Amide I band, and *ν*_as_PO_2_^−^ and *ν*_s_PO_2_^−^ of nucleic acids. The differences between normal cells and ovarian cancer cells were highlighted including (1) a decrease in lipid synthesis (3000–2800 cm^− 1^) in ovarian cancer cells; (2) lower amount of phospholipids in cell membrane (1741 cm^− 1^) in cancer cells; (3) the different proportion of the band intensity of ~ 1454 and ~ 1400 cm^− 1^ in normal cells and in cancer cells, usually I_1454_/I_1400_ ≥ 1 for normal cells and I_1454_/I_1400_<1 for cancer cells; (4) an increase in protein amount (Amide I, Amide II and ν_N-H_ of proteins) in cancer cells.

These results were then verified by tissue samples from 12 ovarian cancer patients. FTIR spectra were compared and analyzed between normal tissues and the corresponding cancer tissues. However, there was no significant difference between normal and malignant tissues at the band intensity of 1741 cm^− 1^. The spectra features of protein (3300 cm^− 1^ and 1544 cm^− 1^) were also not consistent with the cell line results. The intensity ratios of 1454/1400 were still lower in malignant tissues and higher normal tissues; however, *I*_1454_/*I*_1400_>1 or <1 was not the accurate criterion to distinguish normal and cancerous tissues. In addition, lower levels of nucleic acids (1242 cm^− 1^ and 1086 cm^− 1^) were observed in malignant tissues; however, this result was not summarized by cell line data. To integrate cell line data with tissue sample data, we can conclude that the differences between normal and ovarian cancer tissues were included (1) a decrease in lipid synthesis in malignant tissues; (2) lower amount of nucleic acids in malignant tissues; (3) normal tissues showed higher 1454/1400 ratios than malignant tissues. These results have shown the remarkable spectra differences between normal and ovarian cancer cells/tissues with respect to their intensities of the prominent bands of cellular molecules, reflecting changes in the contents of proteins, nucleic acids and lipids. Cell line data also showed heterogeneity existing between different types of ovarian cancer cells. They differed in the synthesis of lipids and nucleic acids. The secondary structure of protein had also been altered between different types of cancer cells, indicating the appearance of new proteins and the alterations in their conformation and composition [23]. As for the data from tissue samples from 12 ovarian cancer patients, there were 10 serous and 2 clear cell cancer. However, no spectra differences were observed between the two types. This may because the sample size was quite small, and it might not be able to summarize the differences.

## Conclusions

Although the results have considerable significance, more reliable evidences from tissue samples with heterogeneous subtypes are necessary to establish the specific and accurate spectral features that could classify malignant ovarian tissues from the normal tissues, as well as identify the precise type and status of malignant tissues. Spectral absorption modes of several heterogeneous ovarian cancer cell lines and some tissue samples primarily provide a research model and proof of principal that there are differences between EOC and healthy donor epithelium, and that this forms the evidences that further investigation is justified. Based on this, in the further study the FTIR spectrum, or prospectively in combination with other assistant methods, would be a useful diagnostic approach for ovarian cancer.

## Abbreviations

ATR-FTIR: Attenuated total reflection Fourier-transform infrared
CA-125: Serum Cancer Antigen 125
CT: Computed Tomography
DII: Second Derivative
EOC: Epithelial ovarian cancer
FTIR: Fourier Transform Infrared
HGSOC: High-grade serous ovarian cancer
HOSEpiC: Human ovarian surface epithelial cell line
MRI: Magnetic Resonance Imaging
